# Shifts in the Spring Herring (*Clupea harengus membras*) Larvae and Related Environment in the Eastern Baltic Sea over the Past 50 Years

**DOI:** 10.1371/journal.pone.0091304

**Published:** 2014-03-17

**Authors:** Timo Arula, Joachim Gröger, Henn Ojaveer, Mart Simm

**Affiliations:** 1 Estonian Marine Institute, University of Tartu, Pärnu, Estonia; 2 Thünen Institute of Sea Fisheries, Hamburg, Germany; 3 Institute for Biosciences, University of Rostock, Germany; 4 Estonian Marine Institute, University of Tartu,Tallinn, Estonia; Vincent, Laudet

## Abstract

Because of the high management relevance, commercial fish related aspects have often been central in marine ecosystem investigations. The iterative shiftogram method was applied to detect occurrence, type and timing of shifts in the single and multivariate time series linked to the spring spawning herring larvae in the Gulf of Riga (Baltic Sea). Altogether nineteen larval herring and related environmental variables were utilized during the period of 1957–2010. All the time series investigated, either single or multivariate, exhibited one or more shifts with variable type and timing. Multivariate shiftogram based on all time series identified two distinct states (1957–1983 and 1992–2010) in studied variables, separated by a smooth transition period lasting almost ten years. The observed shift was mainly related to hydroclimate and not to phenology or biota. Significantly increased variability was found in larval herring and recruitment abundances after the shift. While the shift in hydroclimate (1985–1991) was followed by the shift in phenology (1991–1997), the shift in biota occurred remarkably later (2003). It is likely that the dynamics in biota were affected by other drivers than those investigated in the current paper.

## Introduction

In general, regime shifts are defined as abrupt changes between contrasting persistent equilibrium states of a system [1]. The regime shift concept was initially developed and used in describing earth and athmospheric processes [2], but also became widely applied in the terrestrial [3] and freshwater sciences [4]. In the marine environment, regime shifts have received substantial attention during the past few decades, after the ocean regime shift was identified in the Northern Pacific in the 1970s [5]. After that, regime shifts have been identified in several marine areas, although the time-period covered in those studies rarely start prior to the 1950s [1], [6]. Since then, the regime shift definition has been subsequently broadened to also include biological systems, among others, describing changes in species abundance, community composition, trophic structure, ecosystem state and functioning, as a response to external drivers and forces affecting marine ecosystems. Such phenomena have been observed and explored in several major ocean basins [7] and at different trophic levels [8], [9].

Most of the papers documenting ecological regime shifts [1], [3], [10] have used annual state indicators, making it impossible to examine potential changes in species phenology. However, the seasonal change in sea surface temperature and the succession of several biological parameters may be influenced by climate and human-induced drivers [11], due to both, direct external factors as well as inducing internal processes [12], [13]. As a result, the functioning of the ecosystem may also be affected [14], [15].

So, most of the regime shifts in marine ecosystems have been studied at large marine ecosystems, regional sea level, or at larger spatial scales [16]; there are only a few examples at local spatial scales and coastal areas [17], [18]. Coastal areas and river estuaries perform important functions in marine ecosystems, such as acting as nursery grounds for several commercial fish species, and providing important goods and services for humans. These regions are under various direct anthropogenic pressures and their dynamics may differ from that of the open sea [19]. Accordingly, one should not ignore sub-regional distinct ecosystems, where the nature and magnitude of processes may differ from that in the open sea [18].

Because of the high management relevance, fisheries-related aspects have been important, if not central components of the marine ecosystem analysis [1]. Both historically and presently, herring (*Clupea harengus membras*) is one of the most important commercial fish in the Baltic Sea and the major commercial fish species in the Gulf of Riga (from here on “GoR”). Its ecology has been studied since the early 1950s [20] including several systematic studies on larvae [21]. This creates a favorable basis for establishing the long-term data series, both observed and modelled [21], [22]. The GoR herring is a separate population in the Baltic Sea characterised by a low growth rate. The population does not perform extensive migrations with only a minor part of the older herring leaving the Gulf after the spawning season but returning afterwards [18]. The extent of migrating individuals depends on the stock size and the feeding conditions in the GoR. Before the 1970s, the biomass of a separate sub-population – autumn spawning herring – was more than tenfold higher compared to present. Their share in landings reached up to 40%, however since then it sharply dropped and remained low until nowadays, with the share being considered as negligible [20].

Several numerical methods are available for detecting regime shifts in ecological time series. These are based, among others, on canonical correlation analysis, chronological clustering, sequential t-tests, bootstrapping techniques, regression analysis with forward selection step, dynamical linear models and non-linear diffusion filtering [6], [23]. Basically, regime shifts can be characterized as sudden changes caused by the nonlinear dynamic characteristics of the underlying process which generated the data. Reliable estimation of the exact dynamic structure, however, requires large data sets sampled at high frequencies, whereas ecological phenomena usually become evident in annual data which are mostly available for only a short time span. As a diagnostic device, the “shiftogram” approach among others also copes with this small sample problem by approximating the nonlinear dynamic structure by linear specifications, *i.e*. a set of linear combinations of continuous and discontinuous terms (dummy variables) that have the flexibility to approximate a wide range of break point types [24]. Then, a sequence of statistical parameter tests are applied in order to detect structural breaks indicating (the timing of) shifts [24]. The results of this analysis are comprehensively displayed in an easily interpreted graphic image (“shiftogram”). Hence, the shiftogram method allows to analyse time series sampled even at low frequency in a more comprehensive and vivid manner as compared to the alternative approaches being listed above.

In this paper we have studied nineteen time series describing the hydroclimate, phenology and biota [25] related to the early life history stages of the GoR spring-spawning herring, to explore the occurrence, timing and type of the shifts. These variables were selected based on earlier investigations evidencing their direct or indirect linkage to the dynamics of the early life history stages of the GoR herring [21], [26]–[29]. Specifically we investigated: i) when the shifts in studied variables occurred (by hydroclimate, phenology and biota), ii) which factors, combinations of factors, affected the observed shift in hydroclimate, phenology and biota, iii) whether the shifts in the hydroclimate and phenology evoked temporal response in the biota. Thus, our analyses are not intended to investigate ecosystem-level regime shifts in the GoR by involving all organism groups and trophic levels. Rather it offers a historical view on the dynamics of the hydroclimate, phenology and biota that describe the dynamics of the early life-history stages of the key fish species in the area – spring spawning herring – in a complex and synthetic manner using iterative shiftogram method.

## Materials and methods

### Ethics Statement

No specific permits were required for the described field studies. This work was done in collaboration with the relevant governmental agencies aimed at enhancing the knowledge base on the dynamics of the early life history stages of the regionally most important commercial fish – herring. The study area is not privately-owned and the field studies did not involve endangered or protected species.

### Study area

The Gulf of Riga (area 16,330 km^2^) is a shallow, semi-enclosed sub-basin in the northeastern part of the Baltic Sea. The GoR receives freshwater from a large drainage area (134,000 km^2^), with major inflow in the southern part of the basin. The average salinity varies from 5.0 to 6.5 PSU with the absence of a permanent halocline. Owing to the shallowness (average depth 26 m) of the bay, the dynamics of the surface and deep-water temperatures are directly coupled to the air temperatures. Due to strong vertical mixing, the water column is generally well-oxygenated (oxygen concentrations >5 ml l^−1^) [30].

Pärnu Bay, located in the northeastern part of the GoR, is an enclosed and shallow (average depth 5 m) sea area covering 700 km^2^. Pärnu Bay is covered in ice throughout most winters, while in the warmest summers, the average surface water temperature may reach ca. 24 °C. The salinity varies from 1 to 7.5 PSU. The hydrographic conditions form under the complex influence of ice conditions, freshwater inputs from the Pärnu River and the water exchange with the GoR, that influence salinity, turbidity and nutrients level in the Bay [30].

### Data collection

Based on data availability, we compiled time series of nineteen variables (TS), that describe the ecology of spring-spawning herring's early life history [13], [21], [25], [27], [28], [31], [32]. We demonstrate an approach involving variables by the hydroclimate (1–7), phenology (8–13) and biota (14–19). All the variables were averaged to one value per year for the period of 1957–2010. A summary of the variables used, their units and measurement details, together with the source, is given in Table 1.

**Table 1:** Description of time series.

Variable	Abbreviation	Time	Measure-ment unit	Source	Number of missing years with replacement statistics (n, Rho, p)
1. Winter air temperature	Winter	January- March	°C	EMHI*	-
2. Timing of ice retreat	Ice retreat	Annual	Week	EMHI	-
3. Sea surface temperature, spring	SST spring	April	°C	Omstedt, 2011	3; 0.70; **
4. Sea surface temperature, summer	SST summer	May-July	°C	Omstedt, 2011	4; 0.41; **
5. Sea surface salinity, summer	Salinity	May-July	PSU	Omstedt, 2011	4; 0.91; **
6. Pärnu River inflow	River inflow	Annual	Km^3^ *year^−1^	EMHI	-
7. Water transparency	Transparency	May-July	Meter	Original data	2; 0.72; **
8. Herring larvae onset	Onset	May-July	Week	Original data	9; 0.53; **
9. Larval herring retention time	Retention	May-July	Day	[20] updated	4; 0.68; **
10. Timing of maximum abundance of herring larvae	Her timing	May-July	Week	Original data	9; 0,41; **
11. Timing of maximum abundance of *Eurytemora* nauplii	En timing	May-July	Week	Original data	12; 0.60; **
12. Timing of maximum abundance of Eurytemora affinis females	Eaf timing	May-July	Week	Original data	5; 0.77; **
13. Degree of temporal mismatch between larval herring and *Eurytemora* nauplii	Mismatch	May-July	Week	Original data	20; 0.61; **
14. Copepod nauplii, mean abundance	Cn mean	May-July	Ind.*m^−3^	Original data	1; 0.61; **
15. Adult *Eurytemora affinis*, mean abundance	Ea mean	May-July	Ind.*m^−3^	Original data	1; 0.53; **
16. *Eurytemora* nauplii, maximum abundance	Ean max	May-July	Ind.*m^−3^	Original data	10; 0.65; **
17. Female *Eurytemora affinis*, maximum abundance	Eaf max	Summer	Ind.*m^−3^	Original data	5; 0.46; **
18. Herring larvae, mean abundance	Her larvae	May-July	Ind.*10 min^−1^ haul	[20] updated	6; 0.46; **
19. Herring recruitment	Her recruitment	Annual	Number at age 1 (10^3^)	[20], [31]	-

*Estonian Meterological and Hydrological Institute.

**p<0.01.

Description of the variables used in the current study. 1–7: hydroclimate, 8–13: phenology, 14–19: biota. Numbers before every particular category mean their aggregation for PCA analyses. For more detailed description of variables please see the material and methods.

### Hydroclimatic data

Selected hydroclimatic variables (hereafter: hydroclimate), like; air and water temperature, timing of ice retreat, water salinity, river inflow and water transparency [21], [27]–[31] were obtained from different sources (see Table 1) for the period of 1957–2010. Hydroclimate were characterized by the i) sum of the monthly mean of winter air temperatures, ii) monthly mean sea surface water temperature (SST) in spring and summer and iii) sea surface salinity (salinity) in summer [21], [22]. To show the importance of climatic processes in larger scale the data for Baltic Sea Index in winter was separately analysed [33]. Water transparency was measured by Secchi disc simultaneously with larval herring sampling [27].

### Phenology

Phenological variables (hereafter: phenology) were calculated from the seasonal abundance course of copepods and herring larvae. The onset and the end of the larval herring occurrence were calculated from the cumulative sums of weekly abundances, with the points reaching 10% and 90% from the annual sum, respectively [29]. Larval herring retention time was defined as the number of days from the onset to the end of season. Also the timing of the peak abundance of larval herring and its prey (week of the maximum abundance during the sampling season, Table 1) were included in the phenology.

It has been proposed that the survival success of the herring larvae depends on the overlap between the mass occurrence of the predator – larval herring (m_1_) and its first prey – *Eurytemora affinis* nauplii (m_2_). The critical period for the larval herring is described by the “match-mismatch hypothesis”, stating that the survival of fish larvae depends on whether the peak of the first feeding larvae and their prey production match in space and time (see eq. 1). The match-mismatch (hereafter: ‘mismatch’) [26] was calculated as the week (x) of abundance peak of the predator (m_1_) and its prey (m_2_), where under the conditions of the exact match x = 0 [28] (variable 13, Table 1).

Degree of mismatch (x)  = m_1_−m_2_,

### Biota: copepods and herring abundance

Zooplankton samples were collected weekly from May to July from Pärnu Bay during the daylight. The sampling was performed using the Juday net (mouth opening 0.1 m^2^; mesh size 90 μm) integrating the whole water column vertically (6–7 m) and the samples were analysed similarly to HELCOM guidelines [34]. Seasonal (May-July) mean and maximum abundances of the primary prey items for larval herring, the copepod nauplii and adult *E. affinis* [27], were used in the analyses. Prior to obtaining the seasonal maximum abundances of herring larvae and copepods, weekly mean abundances were normalized according to (eq. 2):
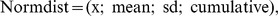
where the **x** is the observation for which we want the distribution, **mean** is the arithmetic mean of the distribution, **sd** is the standard deviation of the distribution and **cumulative** is a logical value that determines the form of the function.

The sampling of fish larvae was performed weekly, mostly from May to July during the daylight. In some occasions, in the end of July herring larvae were still abundant, therefore sampling was continued in August. The samples were taken with a Hensen larval fish trawl (mouth opening d = 80 cm, mesh size 500 μm, codend 190 μm) by 10-minute hauls with speed of ca 2 knots. The collected larvae were immediately preserved in a 4% formaldehyde seawater solution. Altogether 577,540 herring larvae were determined from 3,514 different hauls during the 1957–2010. The data on the 1 year-old fish (recruitment) of the Gulf of Riga herring stock in the 1977–2010 were obtained from [35]. For the years 1957–1976 recruitment estimates originate from [21] (18–19, Table 1).

### Missing value replacement procedure

Most of the variables studied here contain missing values. Although the shiftogram method works with the incomplete time series, in case a structural break (shift) would occur in this position, shift detection is not sufficiently reliable. However, we do not base our study soleley on observed single variables, but mainly on multivariate scores derived from 1^st^ principal components (PCs) of PCA, which reflect specific levels (hydroclimate, phenology, biota). As PCAs require filling-in the missing values, we had to decide upon a method to be the best one in practice for replacing these [36], [37]. The detailed description on the method of replacing missing values in the single time series is provided in Text S1 and Figure S1.

### Statistical analyses

**Figure 1:**
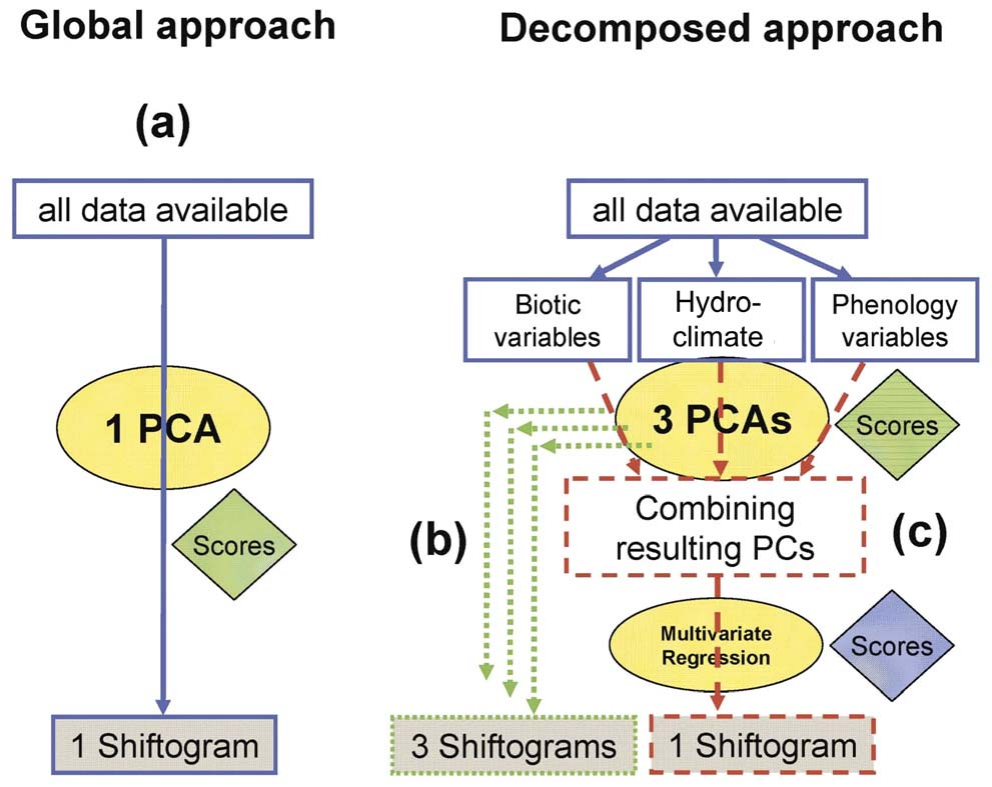
The analysis algorithm (a) for the global approach by applying a PCA based on all variables and generating one shiftogram using the resulting PC1 only, (b) for the decomposed approach by applying three PCAs (one per each factor grouping) and thus generating three shiftograms based on the three resulting PC1's, (c) for the decomposed approach by additionally combining all PCs produced in (b) using multivariate regression and generating one shiftogram based on the predicted values of PC1 of the biotic PCs (see eq. 3).

The shift detection analyses [24] were conducted on the nineteen variables in three different ways: i) by selecting individual variables separately, ii) by the following sub-sets: hydroclimate, phenology, biota (decomposed approach; Fig 1 b,c), and iii) by involving all nineteen time series (global approach; Fig 1a). In ii) and iii), multiple variables were reduced to a single by principle component analysis (PCA), using only the first principal component (PC1) (Fig 1, a-c) as a shiftogram input.

Hierarchical variable clustering was used to test whether the same variables that explain the largest amount of variation in PC1 are clustered together. The number of clusters were based on scree tests of the corresponding PCA. The scree test in the principal component as well as factor analysis selects only those principal components that explain a substantial part of the variance and are thus represented by large eigenvalues. While this normally holds only for the first few PCs, subsequent eigenvalues would remain more or less on the same low level. It has been proposed to apply the so called elbow criterion in a plot of eigenvalues (y axis) against the number of PCs (x axis) below which the eigenvalues will not change much [38], [39]. Hierarchical variable clustering arranges variable associations in a tree-like (cascading) diagram by combining features of PCA and factor analysis and using cluster methods.

### Constructing a shiftogram

Shift detection algorithm can be summarized as follows [24]: while iteratively moving a potential shift point t_0_ over the time series using a specifically defined structural break model (by incrementing t_0_ by 1 year each step), relevant decision criteria described below are recorded during each iteration. The results are displayed in a compound diagrammatic illustration termed as the shiftogram [24]. As the shiftogram simultaneously displays all the data and outcomes resulting from iteratively searching for a potential shift in the time series, it facilitates the interpretation of the results of the iterative screening process for the detection of shifts in the time series. Hence, our shiftogram consists of the 10 component graphic panels, including: i) plot of the time series (**panel 1**), ii) quality-of-fit plot using the corrected Akaike information criterion (**panel 2**), iii) plot of the empirical first order autocorrelation coefficient of the model residuals, given the particular structural break specification (**panel 3**), iv) *p* value of the first order autocorrelation coefficient from the shiftogram (t-test, **panel 4**), v) *p* value of the statistical test of joint significance of all parameters related to the particular structural break specification (F-test, **panel 5**), vi) power plot to indicate the risk of false no-warning; the larger the power, the lower the risk of false no-warning (power  = 1–β, **panel 6**), vii) *p* value of the statistical test of the pure impulse (F-test, **panel 7**), viii) *p* value of the statistical test of a break in slope (F-test, **panel 8**), ix) *p* value of the statistical test of identical levels before and after the shock (ANOVA F-test, **panel 9**), and x) *p* value of the statistical test of the variances before and after the shift (Levene-s test on homoscedasticity, **panel 10**). As a statistical rule of thumb, the size of the window, which is set to 5 years in this case, is required not to exceed 20% of the length of the time series. However, to detect the shifts, only panels ii), v) and vi) aid in localizing the position of the shift in the time series. All other panels help to characterize the type of the shift and which features of the time series have been changed.

## Results

### Interannual dynamics in single variables

**Figure 2:**
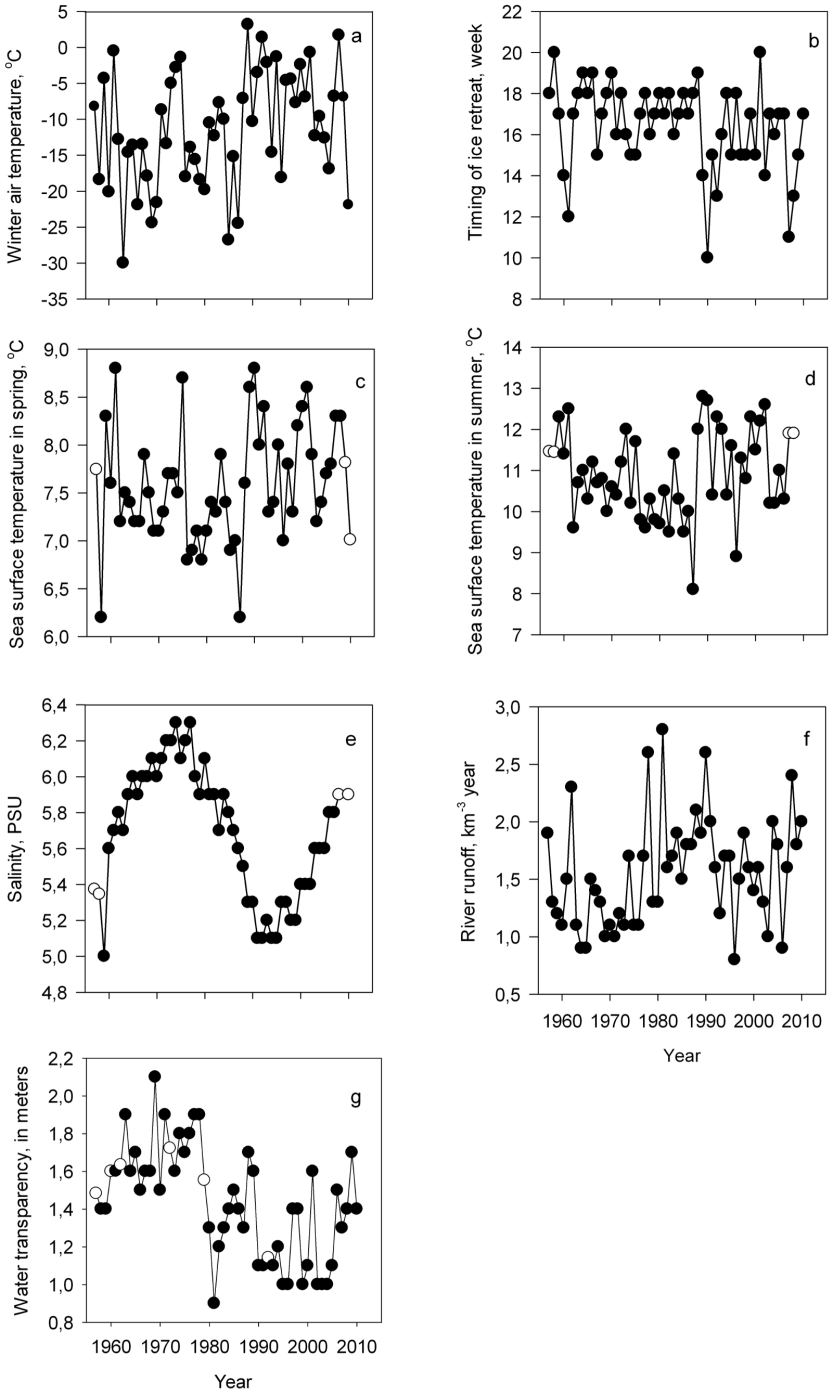
Long term dynamics of hydroclimatic variables as winter air temperature (a), timing of ice retreat (b), mean sea surface temperature in spring (c), mean sea surface temperature in summer (d), mean sea surface salinity in summer (e), mean annual river runoff (f), water transparency in summer (g). Empty dots denote the year when missing value replacement procedure was applied.

In general, winters have become warmer and the ice retreat shifted earlier over time (Fig. 2). Spring SST was generally low until the late 1980s and have increased since then. The decreasing trend in the summer SST reversed in the late 1980s (Fig. 2). Salinity increased until the mid-1970s (up to 6.2 PSU), followed by a decline until the mid-1990s (around 5.1 PSU), and increased recently again. River inflow has gradually, but remarkably increased from the 1960s to the end of the 1980s and dropped since early 1990s. Water transparency displayed generally higher values in the 1960s and 1970s and declined markedly by the early 1980s, exceeding seldomly 1.5 m since then (Fig. 2).

**Figure 3:**
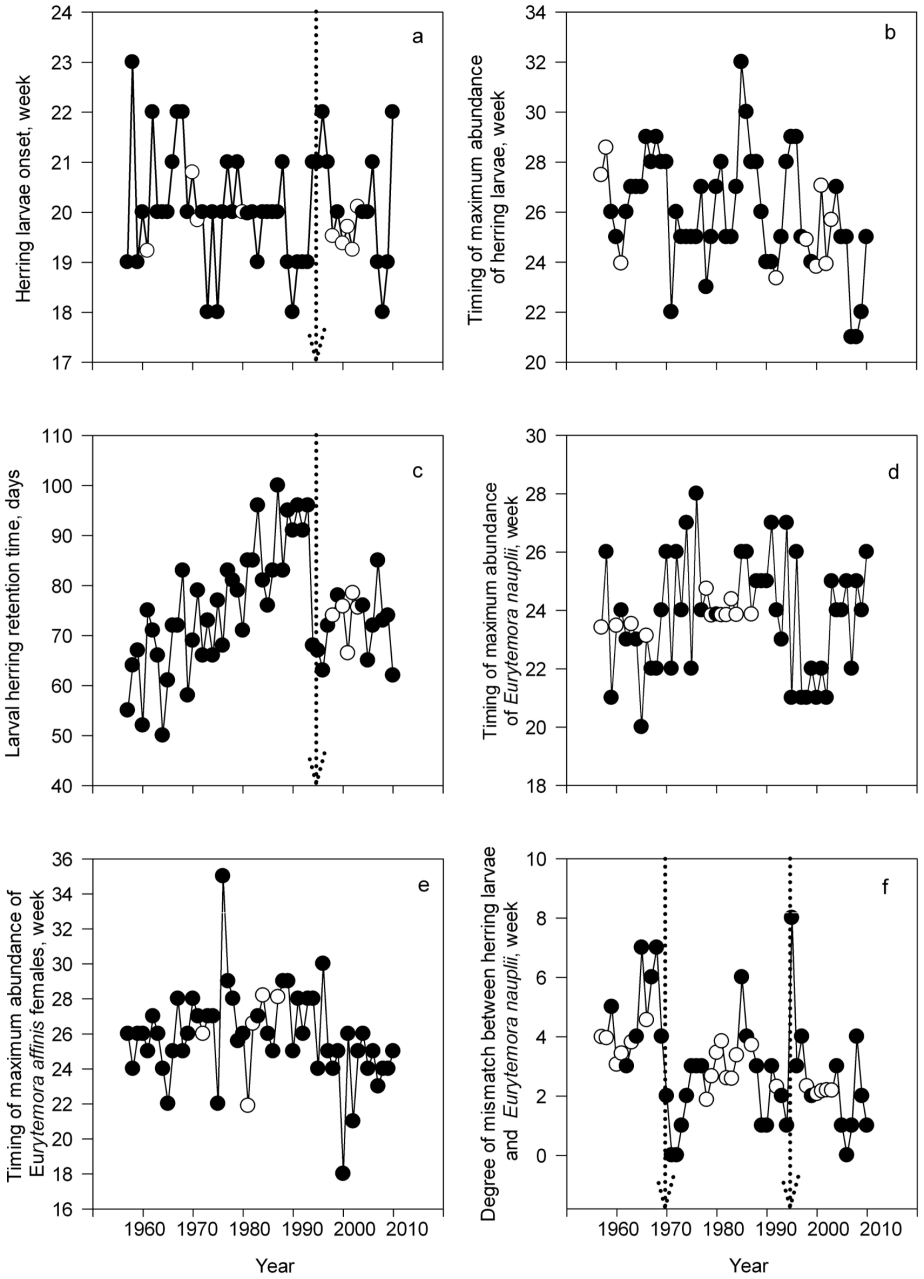
Long term dynamics of phenological variables as larval herring onset (a), timing of maximum abundance of herring larvae (b), larval herring retention time (c), timing of maximum abundance of *Eurytemora affinis* nauplii (d), timing of maximum abundance of *Eurytemora affinis* females (e), degree of mismatch between the timing of maximum abundance of herring larvae and *Eurytemora affinis* nauplii (f). Dotted lines indicate the position of the shift detected in single variables by shiftogram analyses. Empty dots denote the year when missing value replacement procedure was applied.

The onset of herring larvae has varied between weeks 18 and 23 (Fig. 3). The timing of the peak of herring larvae has displayed substantial varibility over time (weeks 21–32), being the most delayed in the late 1980s (Fig. 3). The larval herring retention time increased substantially since the late 1950s until the early 1990s. Since the early 1990s, the retention time remained at the level similar to that in the late 1960s and early 1970s (60–75 days; Fig. 3). Degree of mismatch between herring larvae and its prey decreased over the study period, displaying lowest mismatch in the 1990s (Fig. 3).

**Figure 4:**
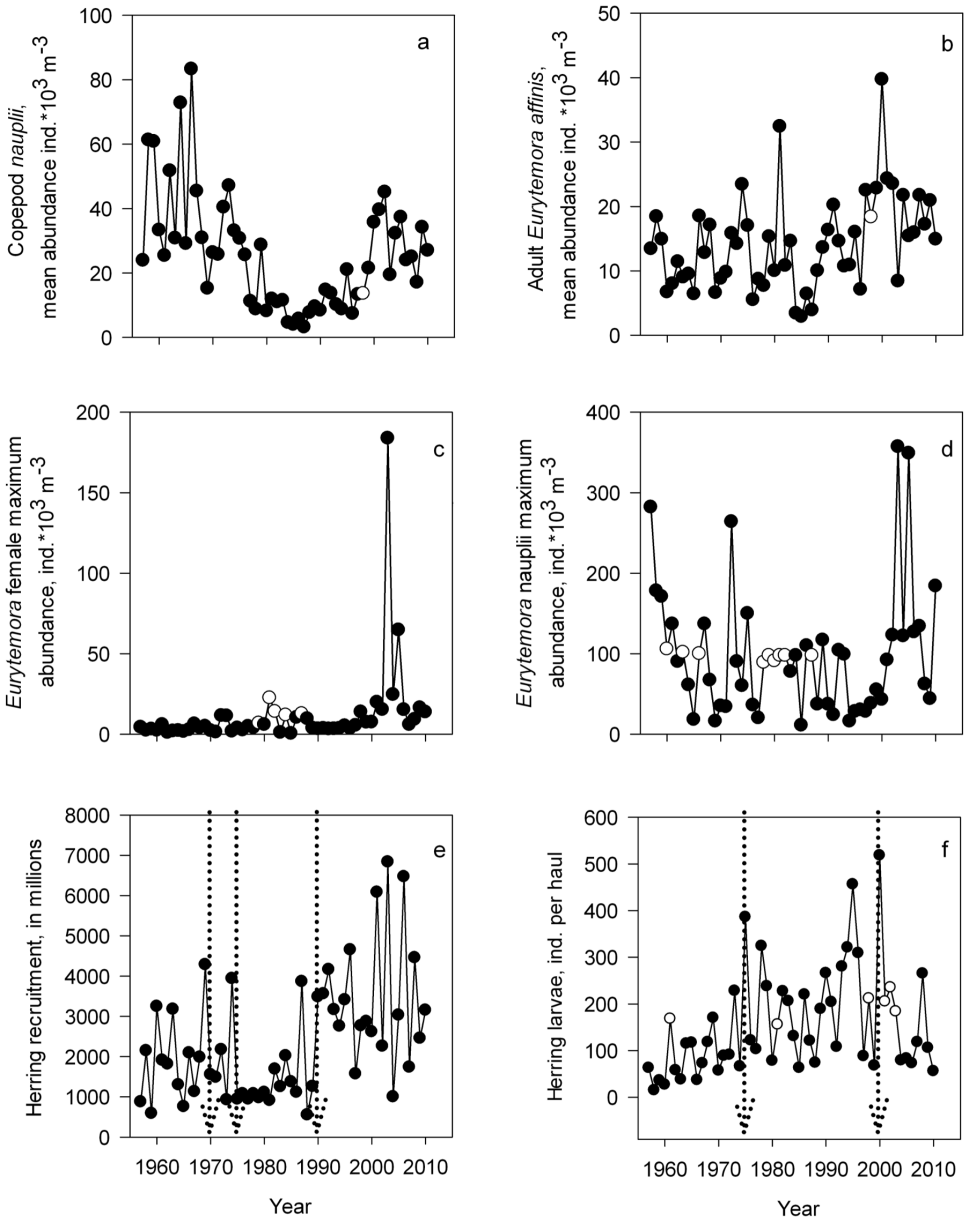
Long term dynamics of biotic variables as mean abundance of copepod *nauplii* (a), mean abundance of adult *Eurytemora affinis* (b), maximum abundance of female *Eurytemora affinis* and nauplii (c), number of herring recruitment (d), mean abundance of herring larvae (e). Dotted lines indicate the position of the shift detected in a single variables by shiftogram analyses. Empty dots denote the year when missing value replacement procedure was applied.

Abundance of copepod nauplii has varied highly over time and is presently about two times lower than in the early 1960s (Fig. 4). Abundance of the adult *E*. *affinis* was less variable compared to copepod nauplii and exhibited increasing trend since the 1980s (Fig. 4). Annual dynamics of the maximum abundance of copepod nauplii and *E*. *affinis* did not show evidence of any long-term pattern and the highest maximum values of both groups were recorded in the early 2000s (Fig. 4). Herring recruitment showed relatively high values since the early 1990s with the highest abundances over time (Fig. 4). Abundance of herring larvae was very low in the beginning of the observation period, but reached the highest values on record by the end of the 1990s. Increase in abundance was accompanied by a larger interannual variability. In the 2000s, the abundance of herring larvae decreased again, and remained at the level of 1960s and the 1970s (Fig. 4).

### Shifts in single variables

First we studied the shifts in the following single variables: the average abundance of herring larvae and recruitment, the retention time and onset of herring larvae and the mismatch between larvae and their prey. Onset and retention time exhibited only one shift, which occurred in the mid-1990s. In both cases, the shift pattern was level-changing. After the shift, the retention time shortened and the onset occurred one week earlier. Two shifts were recorded in herring larvae abundance (impulse-like type; in 1975 and 2000), and in the mismatch between herring larvae and *E. affinis* nauplii (break in the slope type; late 1960s and early 1990s, Table 2). Three shifts (all variance-changing type) were recorded in the herring recruitment abundance: in 1969, 1974 and 1989 (Table 2).

**Table 2:** Timing and types of shifts.

Variable	Timing of shift	Shift type
Larval herring onset	Mid-1990s	Level-changing
Larval herring retention time	Mid-1990s	Level-changing
Degree of temporal mismatch between larval herring and Eurytemora nauplii	Late 1960s, mid-1990s	Break in the slope
Larval herring mean abundance	Mid-1970s, 2000	Impulse-like
Herring recruitment	1969, 1974, 1990	Variance-changing
Hydroclimate	1989	Level-changing
Phenology	Around 1970, mid-1990s, 2003	Slope-changing
Biota	2003	Impulse-like

Timing and types of shifts estimated from the shiftograms of: larval herring mean abundance, herring recruitment abundance, larval herring onset, larval herring retention time and degree of mismatch between larval herring and *Eurytemora* nauplii, hydroclimate, phenology and biota. For principal characterization of the “shift type” (column 3) please see the material and method section ‘*Constructing a shiftogram*’ or [24].

**Figure 5:**
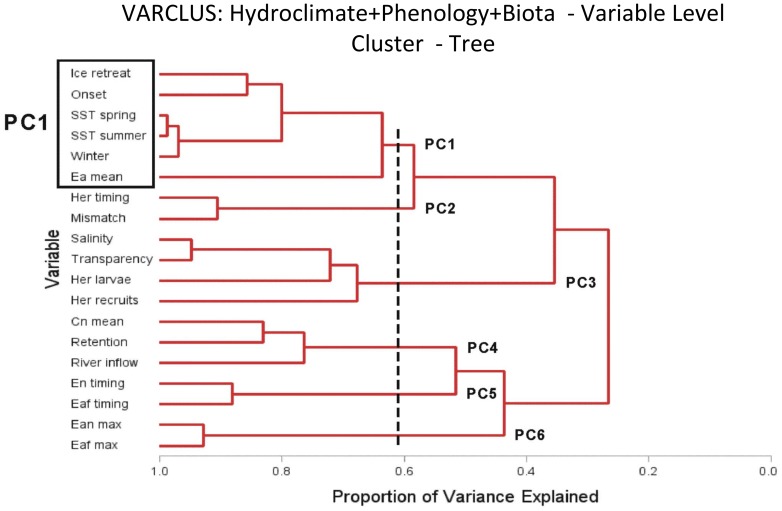
Cluster-tree combining most closely related variables estimated from scree test at Eigen values. Closest variable groups are indicated with the vertical line crossing the x-axis. In x-axis the proportion of variance explained within the formed single group is presented.

Hierarchical clustering identified six similar clusters in studied variables (Fig. 5, see vertical line at 0.6). Most of the variables in the largest cluster characterise the thermal regime (winter air temperatures, ice retreat time, SST in spring and summer). In addition, onset of the herring larvae and average abundance of adult *E. affinis* belong to this group. Two clusters were formed by phenological variables; a first consisting of the mismatch between larvae and prey, and the timing of the peak larvae, and a second consisting of the timing of the seasonal peak larval herring prey.The average abundance of both herring larvae and recruitment were grouped together with salinity and water transparency, while larval fish retention time was in a cluster together with abundance of nauplii and river inflow. The maximum abundance of *E. affinis* females and nauplii formed a separate cluster. A correlation matrix between the variables used in the present study is presented as a Table S1.

### Shifts in hydroclimate, phenology and biota

To identify the contribution of the different variables associated with PC1, only the large eigenvector values of PC1 were considered (values >2 as a thumb of rule) along with highly significant correlations.

Four PCs and clusters were identified by the scree test in the hydroclimate. PC1 explained 47.6% of the total variance and the highest loading on the PC1 has the spring and summer SST and winter air temperatures. These variables were significantly and positively correlated with PC1 (r = 0.86; 0.81; 0.85 respectively, n = 54, p<.01). Salinity and ice retreat were negatively correlated to PC1. Significantly correlated variables were also placed together in the hierarchical variable clustering. Hence, it is likely that these hydroclimatic variables were responsible for one clearly expressed shift. The transition zone from the negative to the positive values of PC1 scores had a smooth pattern lasting from the mid-1980s until the early 1990s, the centre located approximately in 1989. The other minor shift was evident in the early 1960s, but due to short data coverage prior to the early 1960s, reliable conclusions cannot be made.

Three PCs and clusters were identified in species phenology. PC1 explained 34.4% of the total variance and best correlating variables are the onset time of herring larvae, timing of the maximum abundance of herring larvae and the mismatch between herring larvae and their prey. The correlations of the same variables to PC1 were r = 0.76; 0.87; 0.69, respectively (n = 54, p<.01). All other variables explained only a marginal part of variation in PC1. Based on PC1 scores of the phenological variables, two shifts can be identified: one in the 1970s and the another in the mid-1990s.

Four PC1s and clusters were identified in the biota. PC1 explained 30.7% of the total variance, on which the highest loadings were assigned to the recruitment abundance and maximum values of *E. affinis* females and nauplii, with strong and significant correlations to PC1 (r = 0.54; 0.84; 0.87, respectively, n = 54, p<.01). In contrast to the hydroclimate and phenology, biota remained relatively stable over the studied time period and displayed only a single shift in 2003.

**Figure 6:**
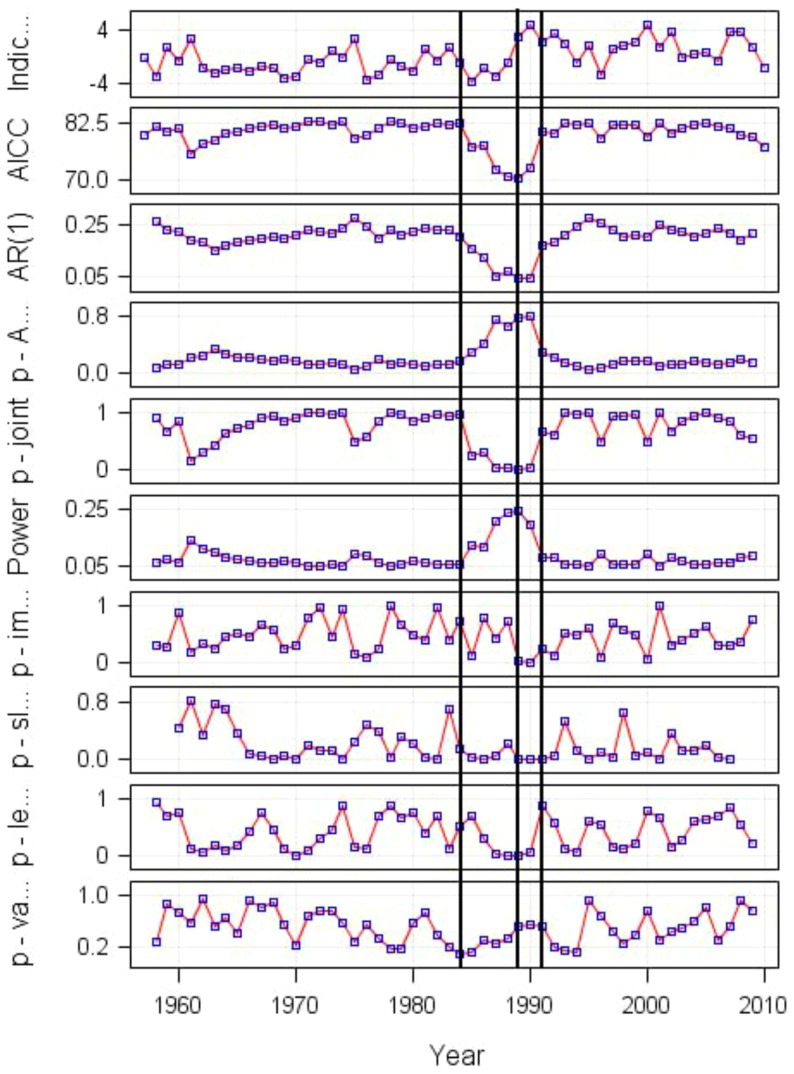
Shiftogram based on PC1 scores derived from all nineteen variables. The vertical lines indicate the position of shift and abbrevations in the Y-axis are from the top: i) plot of the time series analysed (Indic.), ii) quality-of-fit plot (AICC), iii) empirical first order autocorrelation coefficient of the model residuals (AR(1)), iv) *p* value of the first order autocorrelation coefficient (p-A.), v) joint significance relating all parameters (p-joint), vi) power plot to indicate the risk of false no-warning (Power), vii) statistical test detecting the impulse like shift (p-im.), viii) statistical test detecting the break in slope (p-sl.), ix) statistical test detecting identical levels before and after the shock (p-le.), and x) statistical test detecting the variance before and after the shift (p-var.). For details please see the material and method section ‘*Constructing a shiftogram*’ or [24]

**Figure 7:**
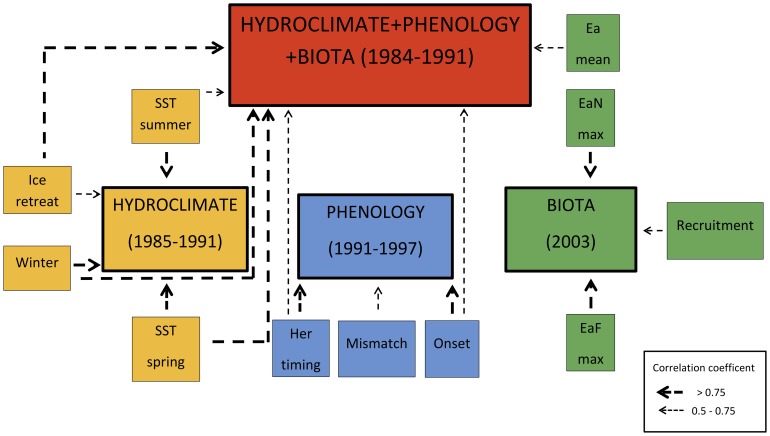
Flow-chart for schematic representation of significant factors that contributed to shifts in the Gulf of Riga for the period of 1957–2010 by different sub-sets (hydroclimate, phenology, biota) and jointly for the all nineteen variables (hydroclimate+phenology+biota). Years referred under different sub-sets indicate timing and duration of a shift. Arrows indicate the suggested causal link between the discriminated categories. All individual factors displayed by sub-sets contributed significantly to the shift in particular sub-set while the underlined parameters contributed significantly to a shift in time series pooling all nineteen variables. Abbrevations in figure are; SST spring and summer: sea surface temperatures in spring and summer, ice retreat: timing of ice retreat, winter: winter air temperature, her timing: timing of maximum abundance of herring larvae, mismatch: degree of temporal mismatch between larval herring and *Eurytemora* nauplii, onset: herring larvae onset, Ea mean: adult *Eurytemora affinis*, mean abundance, EaN max: *Eurytemora* nauplii maximum abundance, EaF max: female *Eurytemora affinis* maximum abundance, recruitment: herring recruitment.

Only one shift was evident in PC1 scores when all nineteen variables were pooled together. Six clusters were identified with PC1 explaining 26.5% of the total variance. Most of the variation in the PC1 was contributed by hydroclimate, such as winter air temperature, SST in spring and SST in summer, but also abundance of herring larvae and *E. affinis*. All these five variables were significantly positively correlated to PC1 (r = 0.84; 0.83; 0.73; 0.47; 0.61, n = 54, p<.01). Timing of the ice retreat, timing of the herring larvae peak abundance and onset were negatively correlated to PC1 scores (r = −0.75; −0.65; −0.66 respectively, n = 54, p<.01). The contribution of all other variables to PC1 scores was not significant. The timing and type of the shift corresponded to that found in the hydroclimate. All key statistics (i.e., AICC value, values of joint significance, and power plot values) indicated the shift in 1989 with a smooth transition period during 1984–1991 (Figs. 6 and 7).

**Figure 8:**
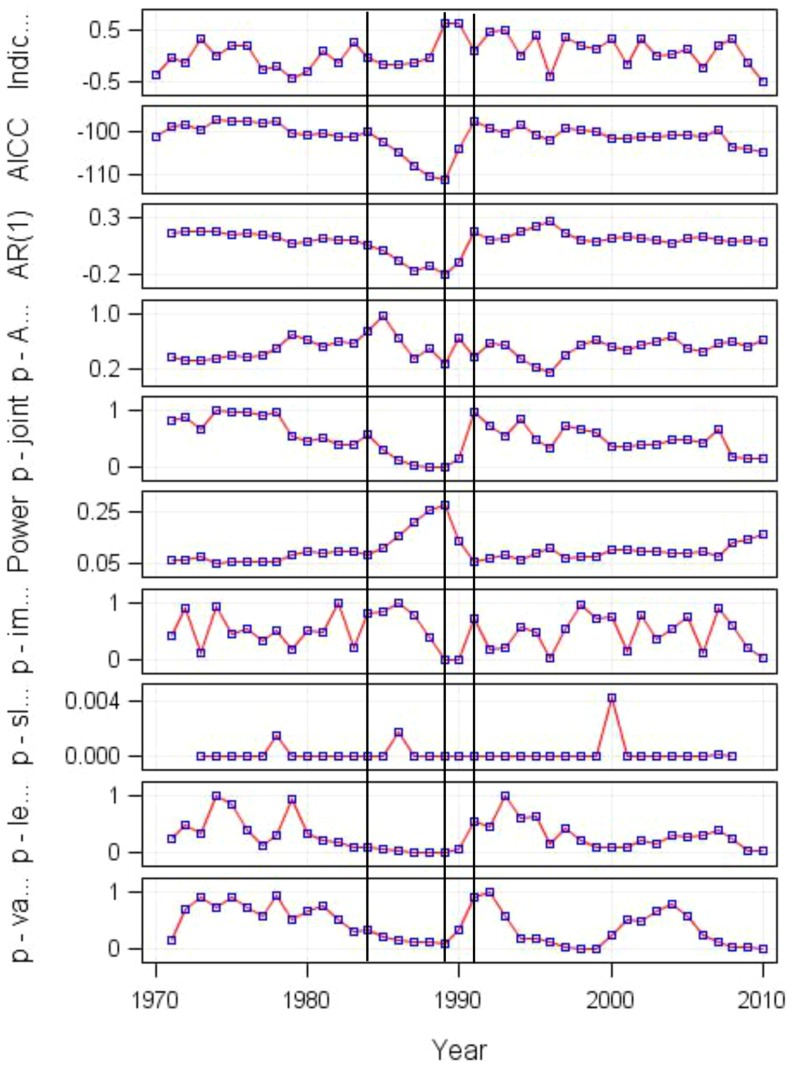
Shiftogram based on Baltic Sea Index in winter. The vertical lines indicate the position of shift and abbrevations in the Y-axis are from the top: i) plot of the time series analysed (Indic.), ii) quality-of-fit plot (AICC), iii) empirical first order autocorrelation coefficient of the model residuals (AR(1)), iv) *p* value of the first order autocorrelation coefficient (p-A.), v) joint significance relating all parameters (p-joint), vi) power plot to indicate the risk of false no-warning (Power), vii) statistical test detecting the impulse like shift (p-im.), viii) statistical test detecting the break in slope (p-sl.), ix) statistical test detecting identical levels before and after the shock (p-le.), and x) statistical test detecting the variance before and after the shift (p-var.). For details please see the material and method section ‘*Constructing a shiftogram*’ or [24]

The main conclusions drawn above are supported by the results obtained from the shiftogram analysis of the winter Baltic Sea Index (BSI): all important statistical characteristics indicate a strong climate effect for the shift in 1989 (also AICC minimum). However, the AICC panel also indicates that gradual change began already in 1984 (Fig. 8). This coincides well with the results obtained above (Figs. 6 and 7). Obviously the BSI-„split marks“ occurred in the same years (1984, 1991) as in case of the PC1 time series involving all nineteen variables. This correspondence was also confirmed by correlation analysis between BSI and PC1 (Pearson r = 0.77, p<.0001).

## Discussion

One of the most often used variable related to climate is temperature, which may influence fish populations in various ways, like reproduction, growth, migration patterns and phenology. It may operate either directly through metabolic and reproductive processes or indirectly by shifting temporal match between prey and predators [12]. We have identified two distinct periods in the hydroclimate conditions with major influence from several temperature-related variables (SST in spring and summer, winter air temperature and timing of ice retreat). Interestingly, other parameters, like river inflow and water transparency had only insignificant effects. Probably, as a result of the changed thermal conditions, several principal changes in species phenologies and abundances appeared. Earlier results have also suggested the key role of climate for regulating herring larvae and recruitment abundance dynamics during various periods in the Gulf of Riga [21].

Species phenologies are rarely considered in studies relating local climate processes and marine ecosystem dynamics, while there are several examples from the inland waters. For instance, a long term analysis of a Dutch lake revealed that clear water phases appeared about one week earlier in the case that temperatures during the first three months of the years was one degree higher ([3],references therein). As a results, the peak of zooplankton occured about two weeks earlier compared to colder winters. There are additional evidences suggesting a linkage between climatic events in the North-Atlantic and changes in seasonal development of various trophic levels like phyto- zoo- and ichthyoplankton, e.g. [13], [29].

As individual species respond differently to changed environmental conditions, ecosystem function might be affected through altered predator-prey relationships, also known as match-mismatch hypothesis [12], [13]. These commonly occur in response to changes in phenology, when rising temperatures cause the temporal advancement of the reproduction of the prey, while the reproduction of the predator remains unaffected [12], [13]. We showed that shifts in phenology occurred a few years later than the shift in the hydroclimate and this might indicate climate impact on the phenology of herring larvae and copepods. Thus, resulting from warmer winters and increased SST in spring and summer, earlier onset and shortened retention period of herring larvae, together with an improved temporal match between herring larvae and their prey was observed. However, the first abrupt change in the phenology of larval herring and copepod at the beginning of the 1970s seems to be independent of the climate. Thus, our paper confirms that inclusion of phenology provides an additional valuable dimension for detection of changes in the marine ecosystems. Altered phenologies may induce concomitant changes in match between prey and predator abundance, and may thus pose further impact on the size of the commercial fish species [28]. Therefore, inclusion of phenology into the long-term marine ecosystem studies has also strong practical management considerations related to better adaptation and mitigation of climate-induced shifts in the size of commercial fish populations [21], [40].

While the species phenology was mainly affected by variables related to herring larvae, maximum abundance of the *E. affinis* adults and its nauplii together with recruitment abundance were the major factors causing the shift in biota. It appears that the shift in the biota occurred about a decade later than the shift in in phenology. Apparently, factors contributing to this shift responded with a long delay to changes in hydroclimate and phenology, or remained unaffected by the factors studied. However, in contrast to the hydroclimate and phenology, the biota remained relatively stable over the time period and displayed only one single abrupt shift in the early 2000s. This might be related to the internalities and resilience of the Gulf of Riga's pelagic ecosystem comprising of only a few brackish copepods and one abundant, well-adapted pelagic planktivorous fish and basically an absence of abundant marine predatory fish species like cod [35].

The shiftogram, pooling all nineteen variables, identified two distinct periods (1957–1983 and 1992–2010), separated by a smooth transition period. Compared to the results from other studies, it is a relatively long transition period for a shift. The observed shift was mainly governed by hydroclimate, while phenology and biota were less important. While the existence and timing of the regime shift was evidenced by previous studies [17], evidence on the ecosystem regime shifts with different timings in the open and coastal areas of the Gulf of Riga also exist (e.g., in 1976/1977; 1997/1998) [17], [18]. It should be noted that all these studies has different approach and data included, thats why they are not comparable with what is presented here. These shifts are not evident in the current analysis, although there seems to be a relatively weak shift in the 1970's. In general, timing of the shift found in the present study coincide with similar events observed in other areas, e.g. the Canadian Eastern Scotian Shelf [41], the U.S. Continental Shelf [42], the North Pacific [43], the North Sea [44], [45] and the Baltic Sea [7], while the pattern of observed shift may differ between areas.

As climate may modify the timing of important behavioral events of organisms [13], [29], we have assumed climate effects to be manifested in the following order: hydroloclimate → phenology → biota. When separately studying additional climatic variable (BSI winter) outside the GoR, that have not been applied in multivariate sub-sets, the shift occurs exactly in the same time as in the shiftogram pooling all variables. This confirms again the prevalence of climate processes that induce the shifts in local hydroclimate and phenology in a rather small and separately located sub-basin of the Baltic Sea. In addition, changes in species phenology may occur not only as a response of bottom-up, but also due to top-down processes, or a combination of both [4]. Hence, both the bottom-up, like climatic or productivity changes, and the top-down processes might be acting together to cause changes in marine ecosystems [7]. This might at least partly explain why phenology of herring prey exhibited different long-term patterns than that of the herring larvae, as there are other abundant predators in the system besides herring, that also consume copepods [46], [47], for which we lack long-term quantitative data.

The major conclusions of the current work is that shifts in hydroclimate and phenology did not evoke a temporal response in the biota. This contrasts the other studies in the open Baltic Sea, where the major changes in hydroclimate were reported to induce bottom-up modifications at different trophic levels [7]. However, our results might not be completely comparable with other studies in this field, essentially because of: i) the different statistical approach, ii) purpose and scientific approach, iii) composition of the main key species in the system (no abundant presence of marine predatory fish), iv) substantially expanded temporal coverage of the study and v) the number of variables (including lack of information on several important trophic levels, e.g. phytoplankton and benthic invertebrates). Such inconsistency in the various layouts certainly points to further needs, suggesting a continuation of process-oriented studies, that could identify the mechanisms responsible for the changes in the variables, studied separately: hydroclimate, species phenology and biota [48].

## Supporting Information

Figure S1 **The flow chart with e concept of the missing value replacement algorithm using the proposed iterative ARIMAX technique ( =  intervention function).** (a) The “response process” indicates the disrupted target variables containing the missing values to be substituted by predictions from the ARIMAX model ( =  endogenous variable). (b) The “highly corresponding information” is represented by one external variables ( =  exogenous variable) identified to be strongly correlated with the target variable which need to be corrected. (c) The algorithm is initialized by first substituting all missing values with the same arithmetic mean calculated from the disrupted target variables. (d) The next step is to fit the ARIMAX model and replace the initial means by initial ARIMAX predictions. (e) to (h) While then looping around the previously inserted ARIMAX predictions are substituted by new ARIMAX predictions in each new iteration step. (g) The algorithm stops as soon as the AICC criterion (quality-of-fit criterion) stabilizes at a low AICC value (empirical AICC minimum) to finally give the reconstructed target variables (“reconstructed response process”).
(TIF)

Table S1 **Table of the correlation matrix between the variables used in present study.**
(DOCX)

Text S1 **Missing value replacement procedure.**
(DOCX)
