# Supplementary figures and images for: Shifts in the Spring Herring (Clupea harengus membras) Larvae and Related Environment in the Eastern Baltic Sea over the Past 50 Years
Source: PLoS One. 2014 Mar 17;9(3):e91304. doi: 10.1371/journal.pone.0091304 (PMC3956613; doi:10.1371/journal.pone.0091304)

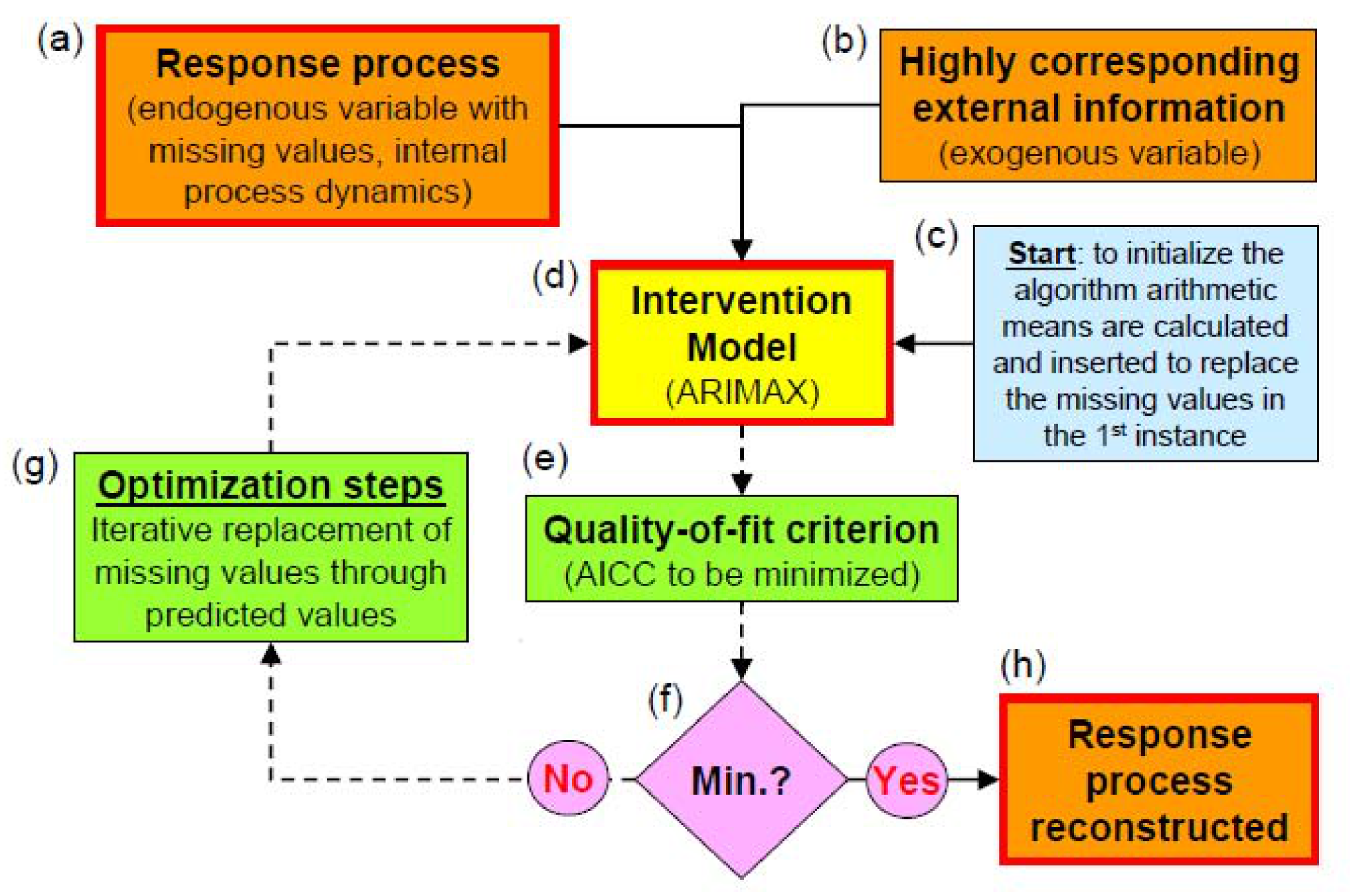

Supplement: Figure S1 — The flow chart with e concept of the missing value replacement algorithm using the proposed iterative ARIMAX technique ( = intervention function). (a) The “response process” indicates the disrupted target variables containing the missing values to be substituted by predictions from the ARIMAX model ( = endogenous variable). (b) The “highly corresponding information” is represented by one external variables ( = exogenous variable) identified to be strongly correlated with the target variable which need to be corrected. (c) The algorithm is initialized by first substituting all missing values with the same arithmetic mean calculated from the disrupted target variables. (d) The next step is to fit the ARIMAX model and replace the initial means by initial ARIMAX predictions. (e) to (h) While then looping around the previously inserted ARIMAX predictions are substituted by new ARIMAX predictions in each new iteration step. (g) The algorithm stops as soon as the AICC criterion (quality-of-fit criterion) stabilizes at a low AICC value (empirical AICC minimum) to finally give the reconstructed target variables (“reconstructed response process”). (TIF) [file pone.0091304.s001.tif]
