# Supplementary material for: Shifts in the Spring Herring (Clupea harengus membras) Larvae and Related Environment in the Eastern Baltic Sea over the Past 50 Years
Source: PLoS One. 2014 Mar 17;9(3):e91304. doi: 10.1371/journal.pone.0091304 (PMC3956613; doi:10.1371/journal.pone.0091304)
Supplement: Table S1 — Table of the correlation matrix between the variables used in present study. (DOCX) [file pone.0091304.s002.docx]

| **VARIABLE** | Her larvae | Recruits | Cn mean | Ea mean | Ean max | Eaf max | Winter | Salinity | Trans-parency | River inflow | Ice retreat | SST spring | SST summer | Retention | Onset | Her timing | En timing | EaF timing | Mismatch |
| --- | --- | --- | --- | --- | --- | --- | --- | --- | --- | --- | --- | --- | --- | --- | --- | --- | --- | --- | --- |
| Her larvae |  | 0.1345 | -.2853 | 0.2349 | -.2190 | 0.0145 | 0.4014 | -.2331 | -.3034 | 0.1044 | -.2917 | 0.2921 | 0.1312 | 0.2428 | -.3051 | -.1550 | -.1287 | -.2405 | -.0302 |
| Recruits | 0.1345 |  | -.1122 | 0.0806 | 0.1380 | 0.4340 | -.0138 | -.2640 | -.2328 | -.1181 | -.0668 | 0.1169 | -.0254 | -.0626 | 0.0178 | -.1317 | 0.1560 | -.0614 | -.2280 |
| Cn mean | -.2853 | -.1122 |  | 0.2716 | 0.2577 | -.0347 | -.0437 | 0.1273 | 0.1746 | -.3162 | 0.1923 | 0.0492 | 0.2503 | -.4950 | 0.1686 | 0.0067 | -.2663 | -.2789 | 0.1028 |
| Ea mean | 0.2349 | 0.0806 | 0.2716 |  | 0.0210 | -.0130 | 0.4734 | -.2248 | -.3772 | 0.2569 | -.2560 | 0.4452 | 0.4051 | 0.0916 | -.1410 | -.3213 | -.3042 | -.5974 | -.1668 |
| Ean max | -.2190 | 0.1380 | 0.2577 | 0.0210 |  | 0.6113 | 0.0178 | -.0229 | -.1769 | -.0446 | 0.0555 | 0.0585 | 0.1503 | -.1572 | -.0255 | -.0648 | 0.0414 | -.1697 | -.2195 |
| Eaf max | 0.0145 | 0.4340 | -.0347 | -.0130 | 0.6113 |  | 0.0065 | -.0396 | -.3083 | -.0649 | 0.0585 | -.0622 | -.0907 | -.0029 | -.0100 | -.0479 | 0.0665 | -.1253 | -.1532 |
| Winter | 0.4014 | -.0138 | -.0437 | 0.4734 | 0.0178 | 0.0065 |  | -.3622 | -.2818 | 0.2502 | -.5767 | 0.7037 | 0.6439 | 0.3135 | -.5333 | -.5277 | -.2964 | -.2314 | -.1633 |
| Salinity | -.2331 | -.2640 | 0.1273 | -.2248 | -.0229 | -.0396 | -.3622 |  | 0.6169 | -.1510 | 0.1773 | -.3149 | -.4091 | -.1747 | 0.0740 | -.0131 | 0.1758 | 0.1121 | -.0326 |
| Transparency | -.3034 | -.2328 | 0.1746 | -.3772 | -.1769 | -.3083 | -.2818 | 0.6169 |  | -.2239 | 0.2047 | -.1563 | -.1417 | -.3009 | 0.0747 | 0.0101 | 0.0668 | 0.2899 | 0.0247 |
| River inflow | 0.1044 | -.1181 | -.3162 | 0.2569 | -.0446 | -.0649 | 0.2502 | -.1510 | -.2239 |  | -.2874 | 0.1500 | 0.0814 | 0.3987 | -.1409 | -.1416 | 0.1532 | -.0238 | -.0667 |
| Ice retreat | -.2917 | -.0668 | 0.1923 | -.2560 | 0.0555 | 0.0585 | -.5767 | 0.1773 | 0.2047 | -.2874 |  | -.6322 | -.4658 | -.3489 | 0.5284 | 0.6426 | 0.1514 | 0.2283 | 0.1846 |
| SST spring | 0.2921 | 0.1169 | 0.0492 | 0.4452 | 0.0585 | -.0622 | 0.7037 | -.3149 | -.1563 | 0.1500 | -.6322 |  | 0.7715 | 0.1439 | -.5818 | -.4629 | -.3185 | -.3221 | -.1569 |
| SST summer | 0.1312 | -.0254 | 0.2503 | 0.4051 | 0.1503 | -.0907 | 0.6439 | -.4091 | -.1417 | 0.0814 | -.4658 | 0.7715 |  | 0.0620 | -.4468 | -.3608 | -.3181 | -.3223 | -.1403 |
| Retention | 0.2428 | -.0626 | -.4950 | 0.0916 | -.1572 | -.0029 | 0.3135 | -.1747 | -.3009 | 0.3987 | -.3489 | 0.1439 | 0.0620 |  | -.3003 | -.2067 | 0.0515 | 0.1270 | -.1773 |
| Onset | -.3051 | 0.0178 | 0.1686 | -.1410 | -.0255 | -.0100 | -.5333 | 0.0740 | 0.0747 | -.1409 | 0.5284 | -.5818 | -.4468 | -.3003 |  | 0.5213 | 0.1173 | 0.1859 | 0.2616 |
| Her timing | -.1550 | -.1317 | 0.0067 | -.3213 | -.0648 | -.0479 | -.5277 | -.0131 | 0.0101 | -.1416 | 0.6426 | -.4629 | -.3608 | -.2067 | 0.5213 |  | 0.1405 | 0.1763 | 0.5620 |
| En timing | -.1287 | 0.1560 | -.2663 | -.3042 | 0.0414 | 0.0665 | -.2964 | 0.1758 | 0.0668 | 0.1532 | 0.1514 | -.3185 | -.3181 | 0.0515 | 0.1173 | 0.1405 |  | 0.5559 | -.3280 |
| EaF timing | -.2405 | -.0614 | -.2789 | -.5974 | -.1697 | -.1253 | -.2314 | 0.1121 | 0.2899 | -.0238 | 0.2283 | -.3221 | -.3223 | 0.1270 | 0.1859 | 0.1763 | 0.5559 |  | -.1051 |
| Mismatch | -.0302 | -.2280 | 0.1028 | -.1668 | -.2195 | -.1532 | -.1633 | -.0326 | 0.0247 | -.0667 | 0.1846 | -.1569 | -.1403 | -.1773 | 0.2616 | 0.5620 | -.3280 | -.1051 |  |

Tabel S1. Table of the correlation matrix between the variables used in present study.
