# Supplementary material for: Shifts in the Spring Herring (Clupea harengus membras) Larvae and Related Environment in the Eastern Baltic Sea over the Past 50 Years
Source: PLoS One. 2014 Mar 17;9(3):e91304. doi: 10.1371/journal.pone.0091304 (PMC3956613; doi:10.1371/journal.pone.0091304)
Supplement: Text S1 — Missing value replacement procedure. (DOCX) [file pone.0091304.s003.docx]

Annex S1. Missing value replacement procedure.

The set of options for replacing missing values is however limited to the following alternatives (including the null option): i) Leaving-in the gaps (null option), ii) Replacing missing values by zeros, iii) Replacing missing values by total means, iv) Replacing missing values by local or running means, v) Replacing missing values using polynomials, vi) Replacing missing values by numerical inter-/extrapolation using, for instance, spline functions, vii) Replacing missing values by means of regression methods, viii) Replacing missing values using intervention functions, ix) Replacing missing values using intervention functions with exogenous variables, and finally x) Repeated replacement of missing values using iterative intervention functions with exogenous variables. However, only the algorithms supporting the idea of reconstructing the internal dynamics of the disrupted time series would allow for an unbiased estimation of appropriate values to fill the gaps; otherwise the analysis would give biased and/or misleading results [[36](#_ENREF_36)]. Given this constraint, options viii) to x) seem to be the most appropriate ones, where option x) indicates an algorithm that has the best statistical properties by at the same time using most of the information sources available. This is the reason why we selected this method as finally the best choice. This approach is designed to focus especially on reconstructing the internal dynamics of the interrupted time series under consideration by at the same time being based on a substantially richer amount of information compared to alternative methodology; it however also works, when no other than the internal information of the interrupted time series is given.

Option x) is an extension of the algorithm proposed by [[36](#_ENREF_36)]. Intervention functions are basically autoregressive integrated moving average (ARIMA) models extended by a dummy coded “break variable” (binary indicator variable) representing the missing value as a structural break which in time series terminology is also called an intervention. We further modified the proposed method by adding corresponding external information (exogenous variable) aiming at an improvement of the prediction to give an ARIMAX model (transfer function). In addition we implemented a series of iteration steps that stop looping around as soon as a performance (quality-of-fit) criterion to be minimized (AICC = Akaike’s information criterion corrected for small samples; see [[37](#_ENREF_37)]) stabilizes by converging a rather small value (AICC-threshold); accordingly the predicted substitutes become iteratively improved. The AICC takes into account changing degrees of freedom. Like other ARIMA models, intervention functions require equidistant time stamps. Hence, to initialize this algorithm, in the 1^st^ instance the mean value of the incomplete time series (endogenous variable) under consideration is calculated and then inserted to replace its missing values. While looping around, these means are then replaced by new predictions of the intervention function in a stepwise manner which per each iteration gets re-estimated. The conceptual algorithm is shown in Fig. 1; it is realized in SAS, Version 9.3.

In addition, we performed a series of simulations to test the performance of our missing value replacement method. Accordingly, we contrasted the performance of our method with that of three alternative standard methods taken from the set of options above: the substitution of missing values (1) by zeros, (2) by means, and (3) by predicted values from regression. To do this, we simulated 12 time series as AR[1,2,3] processes without and from these generated 12 additional time series to correlate with the first ones. Essential part of the simulations was to gradually change the strength of correlation and the degree of error variation (innovation and noise variance, respectively). The 12 correlated time series are used as exogenous input variables for the iterative ARIMAX as well as the regression technique. To be compatible with reality each of the 24 time series contained n=50 values (50 years). To generate data gaps we cloned each of the first 12 AR[1,2,3] time series by at the same time randomly removing 20% of the values per each of the cloned time series through bootstrapping. This allowed us to contrast the complete time series with their incomplete copies and to compare the performance of the four different missing value substitution methods. After value reconstruction through the four different missing value substitution methods, we compared their performance by computing Pearson’s correlation coefficients (r) between the 12 complete AR[1,2,3] time series and their incomplete clones per each method where r = 1 means perfect reconstruction. We repeated this numerical simulation experiment three times.

The numerical simulation experiments revealed that in all cases the iterative ARIMAX method received superior results, on average reaching a correlation coefficient of 0.98 (range 0.06), followed by the regression method with 0.92 (range 0.13), the mean insertion method with 0.88 (range 0.15) and the zero insertion method with 0.46 (range 0.70). In case of the iterative ARIMAX technique the result was almost independent of the strength of correlation of the AR[1,2,3] time series with the corresponding input time series, while that of the regression improved the better the correlation with the corresponding input time series was; it however never reached the performance of the ARIMAX method. The range shows that the results of the iterative ARIMAX method remain very stable over the different time series, whilst that of the other methods appear much wider. In terms of information loss this means, that in case of the iterative ARIMAX method only 4% of the variance is left unexplained, while in case of all other methods 15% (regression), 23% (mean insertion), and 79% (zero insertion) are left unexplained. These findings strongly support and justify using the iterative ARIMAX method.
